# Combining functional weed ecology and crop stable isotope ratios to identify cultivation intensity: a comparison of cereal production regimes in Haute Provence, France and Asturias, Spain

**DOI:** 10.1007/s00334-015-0524-0

**Published:** 2015-03-19

**Authors:** Amy Bogaard, John Hodgson, Erika Nitsch, Glynis Jones, Amy Styring, Charlotte Diffey, John Pouncett, Christoph Herbig, Michael Charles, Füsun Ertuğ, Osman Tugay, Dragana Filipovic, Rebecca Fraser

**Affiliations:** 1 School of Archaeology, University of Oxford, 36 Beaumont Street, Oxford, OX1 2PG UK; 2 Department of Archaeology, University of Sheffield, Northgate House, West Street, Sheffield, S1 4ET UK; 3 Abt. Vor- und Frühgeschichte, Institut für Archäologische Wissenschaften, J.W. Goethe-Universität, Grüneburgplatz 1, 60323 Frankfurt/Main, Germany; 4 Orhangazi caddesi, Kumbaşı yolu no 109, Iznik, Bursa, Turkey; 5 Biyoloji Bölümü, Fen Fakültesi, Selçuk Üniversitesi, Selçuklu, 42075 Konya, Turkey; 6 Institute for Balkan Studies, Serbian Academy of Sciences and Arts, Knez Mihailova 35/IV, Belgrade, Serbia

**Keywords:** Archaeobotany, Weed ecology, Stable isotopes, Agricultural intensity, Neolithic

## Abstract

This investigation combines two independent methods of identifying crop growing conditions and husbandry practices—functional weed ecology and crop stable carbon and nitrogen isotope analysis—in order to assess their potential for inferring the intensity of past cereal production systems using archaeobotanical assemblages. Present-day organic cereal farming in Haute Provence, France features crop varieties adapted to low-nutrient soils managed through crop rotation, with little to no manuring. Weed quadrat survey of 60 crop field transects in this region revealed that floristic variation primarily reflects geographical differences. Functional ecological weed data clearly distinguish the Provence fields from those surveyed in a previous study of intensively managed spelt wheat in Asturias, north-western Spain: as expected, weed ecological data reflect higher soil fertility and disturbance in Asturias. Similarly, crop stable nitrogen isotope values distinguish between intensive manuring in Asturias and long-term cultivation with minimal manuring in Haute Provence. The new model of cereal cultivation intensity based on weed ecology and crop isotope values in Haute Provence and Asturias was tested through application to two other present-day regimes, successfully identifying a high-intensity regime in the Sighisoara region, Romania, and low-intensity production in Kastamonu, Turkey. Application of this new model to Neolithic archaeobotanical assemblages in central Europe suggests that early farming tended to be intensive, and likely incorporated manuring, but also exhibited considerable variation, providing a finer grained understanding of cultivation intensity than previously available.

**Electronic supplementary material:**

The online version of this article (doi:10.1007/s00334-015-0524-0) contains supplementary material, which is available to authorized users.

## Introduction

Agricultural intensity—defined here in terms of labour inputs per unit area of arable land—represents a crucial axis for assessing the scale and productivity of past farming regimes, and their social and wider ecological implications (Gilman 1981; Sherratt 1997; Trigger 2003). An earlier study of the floristic composition and functional ecological profile of weed flora in Evvia, Greece identified a particular set of weed functional attributes relating to soil fertility and disturbance that distinguished intensive ‘garden’ and extensive ‘field’ cultivation of broad beans and other pulses (Jones et al. 1999, 2000). This contrast between high- and low-intensity regimes, framed using discriminant analysis, was applied to another crop type (spelt wheat) and region (Asturias, Spain) as a modern ‘test’ and found to identify intensive cereal cultivation successfully (Charles et al. 2002). Application of this same approach to Neolithic archaeobotanical weed data from central Europe characterised early farming as intensive ‘garden’ cultivation (Bogaard 2004).

Subsequent work on the stable carbon and nitrogen isotope composition of crops grown under a range of experimental and ‘traditional’ regimes identified the δ^15^N and δ^13^C values of cereals and pulses as useful parameters for assessing the contribution of manuring and watering, respectively, to soil productivity (Fraser et al. 2011; Wallace et al. 2013). Intensive fertilisation of crops with animal dung results in ^15^N enrichment due to the volatilisation of lighter ^14^N in ammonia, and hence elevated crop δ^15^N values, while long-term cultivation without manuring results in low crop δ^15^N values (Choi et al. 2003; Bol et al. 2005; Bogaard et al. 2007). Crop δ^13^C values, by contrast, reflect water status due to increasing discrimination against ^13^C when stomata are closed in dry conditions (Farquhar and Richards 1984; Condon et al. 1987; Araus et al. 1999).

There are clear advantages to combining weed ecology and crop isotope determinations in order to assess agricultural practice (cf. Ferrio et al. 2007; Bogaard et al. 2013). Weed ecology is needed to assess the permanence of arable land (Bogaard 2002, 2004), and also provides evidence of soil disturbance levels (due to tillage and weeding). While weeds also give a general index of soil productivity, crop δ^15^N indicates the composition of available soil N (including possible manuring effects, naturally abundant organic matter—in both cases with preferential loss of ^14^N through ammonia volatisation—and the action of denitrifying bacteria under anaerobic conditions—Billy et al. 2010), while crop δ^13^C values reflect water status (resulting from water management as well as natural sources such as precipitation). Stable isotope determinations of crops therefore provide a direct means of assessing the possible contributions of manuring and water management to crop growth, within the overall framework of arable fertility, disturbance and permanence established through weed ecology. Functional ecological attributes and plant stable isotope values are subject to different sources of error and ambiguity; interpretations that incorporate evidence from both approaches are more robust and informative than inferences based on only one form of evidence (Bogaard 2015).

**Fig. 1:**
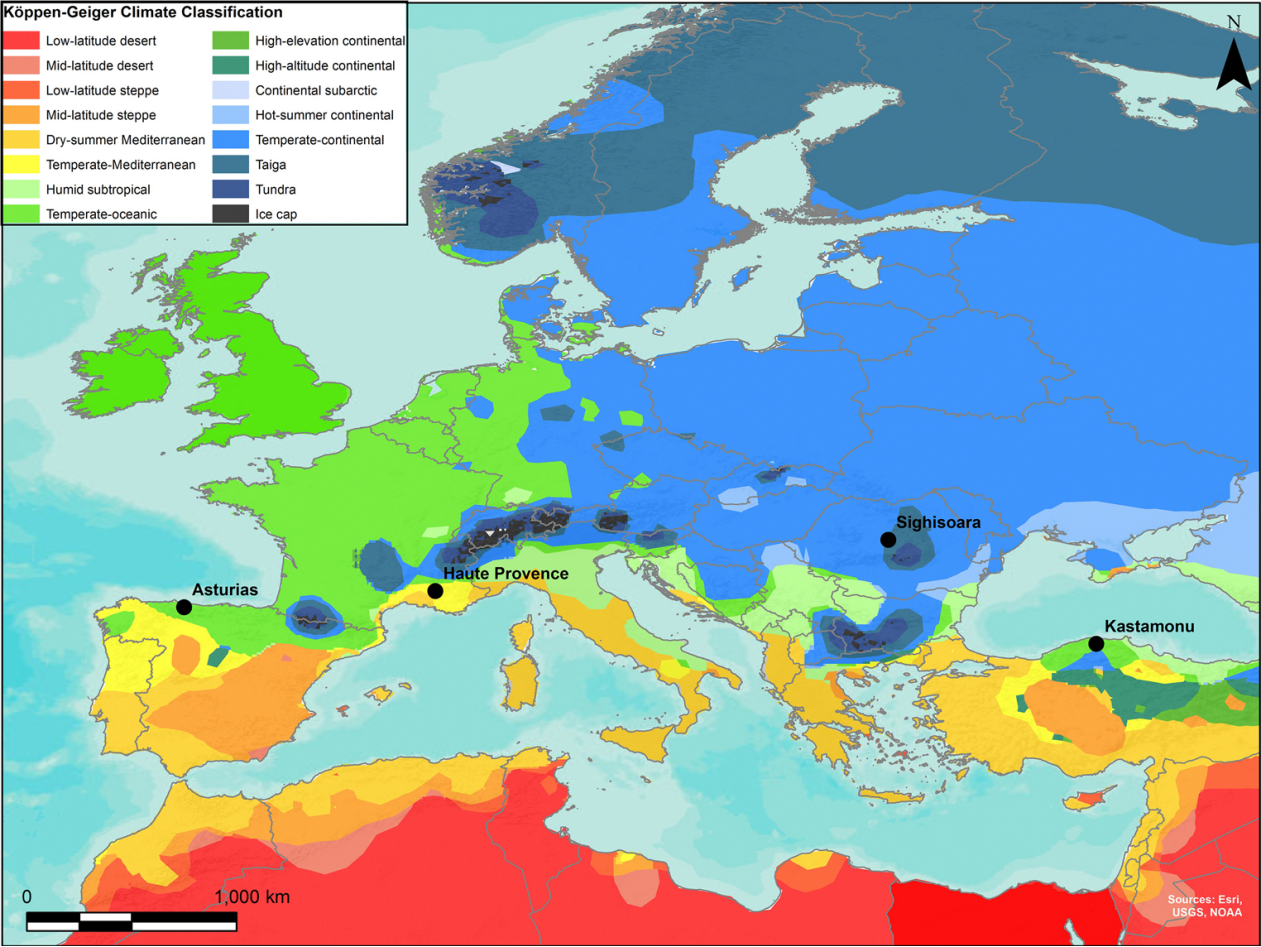
Map showing modern agricultural study locations

The central aim of the present study is to combine weed ecological analysis and crop stable isotope determinations in order to characterize a contrast in intensity between two cereal production regimes in south-western Europe: intensive management of relatively small-scale production in Asturias, Spain (Charles et al. 2002), and larger scale, low-intensity production in Haute Provence, France. The present investigation extends previous studies in two ways. First, while floristic/ecological weed data and crop stable isotope values are available for intensively managed cereals in Asturias (Charles et al. 2002; Fraser et al. 2011), data on organically grown cereals managed with *low* intensity have been lacking. Moreover, since the δ^15^N values of pulses are affected by fixation of atmospheric nitrogen, previous isotopic survey of high- and low-intensity *pulse* cultivation in Evvia (Fraser et al. 2011) cannot be ‘translated’ for *cereals*. A study of large-scale, low-intensity organic cereal production in Haute Provence was therefore conducted in order to fill these gaps. Secondly, the present study seeks to characterize the ecological contrast between high- and low-intensity cereal production systems that lack irrigation, in contrast to more arid systems where supplementary watering is a likely potential component of intensive cultivation. A further objective is therefore to assess the range and causes of variation in crop δ^13^C values where no artificial watering was practised. Moreover, to test the general applicability of the new ‘weed + isotope’ model of cereal cultivation intensity, we apply it to two other present-day cereal regimes: small-scale/intensive cultivation of einkorn and other cereals in the Sighisoara region, Romania, and large-scale/low-intensity cultivation of einkorn and emmer in Kastamonu, Turkey (Fig. 1). Finally, to investigate its archaeobotanical utility, this study includes preliminary reassessment of the intensity of cereal production systems in Neolithic central Europe, building on previous work by Bogaard (2004), through application of the new multi-stranded model.

### The study region in Haute Provence

**Fig. 2:**
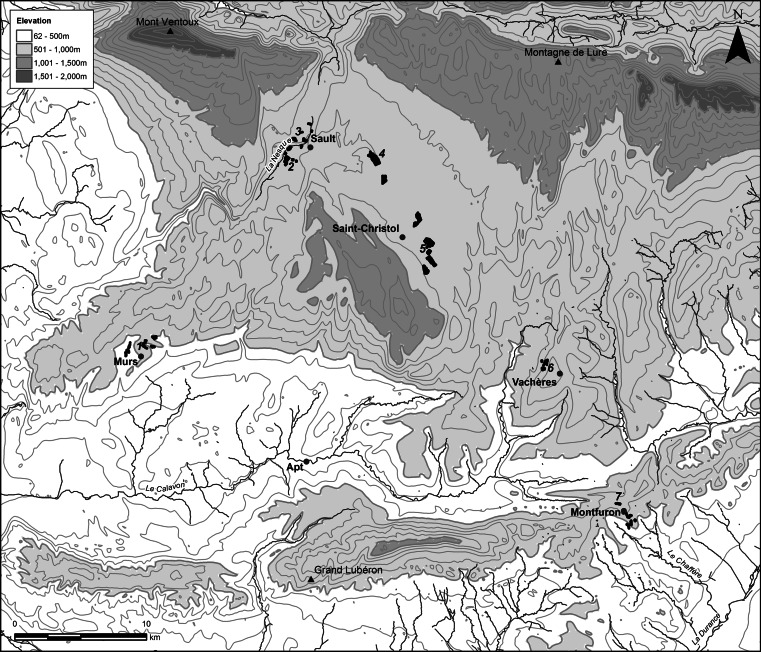
Map showing the study area in Haute Provence, France and the locations of the seven farms; the fields surveyed are indicated in *black shading*

The geographical focus of this study is a region within Haute Provence, south-eastern France, framed by the limestone massifs of Mont Ventoux (the region’s highest peak, 1912 m) and the Montagne de Lure in the north, and the Lubéron massif in the south (Fig. 2). The study area includes two calcareous plateaux (Sault and Albion) at c. 800–1,000 m altitude to the south-east of Mont Ventoux, and more varied ‘molasse’ geology (including calcareous sandstones and clays) and fractured topography in the northern Lubéron (c. 500–700 m).

Climatically, this area occupies a transitional position between the semi-humid Mediterranean coast and oceanic/continental conditions—Mont Ventoux is considered the absolute limit of broadly Mediterranean bioclimate in the region—and experiences persistent cooling by the mistral and transmontane winds (Blondel et al. 2010, 103). The Lubéron range to the south of the study area forms an approximate boundary between the classic mediterranean conditions to the south and oceanic/continental climate further north: in Köppen–Geiger climate classification terms the Sault area is temperate-oceanic (Cfb), while the northern Lubéron area around Apt is temperate-mediterranean (Csb). Average annual precipitation ranges from c. 900 mm around Sault to c. 700 mm around Apt; rainfall around Sault remains high year-round, with peaks in October and May, while rainfall in the southern part of the study area around Apt follows a more mediterranean pattern, being distinctly higher in winter (peaking in October and February) than summer.

A total of 57 crop fields was studied near Sault (c. 800 m altitude), Saint-Christol (c. 800–1,000 m) and Murs (c. 500 m) in the Vaucluse, and near Vachères (c. 700 m) and Montfuron (c. 500 m) in Alpes-de-Haute Provence (Fig. 2). The fields belonged to seven organic farms; contacts to producers were established through the chambre d’agriculture in Sault and the parc naturel du Lubéron. The farms ranged from relatively large-scale holdings (240–300 ha) on the plateau d’Albion, comprised of fields averaging 8–9 ha each, to smaller farms of <100 ha with fields averaging c. 1–2 ha (Table 1). Crop rotation was practised on all farms and considered crucial for maintaining soil fertility in the (virtual) absence of manuring (see below). At four of the farms, rotation regimes featured 8–10+ years of [perennial] aromatic crops (especially *Lavandula angustifolia*, lavender, and *L. hybrida*, lavandin) followed by a shorter period of annual crops including cereals, forage legumes and sometimes pulses; in three cases *Triticum monococcum*, einkorn, was grown as an initial annual crop following aromatics (Table 1). At the remaining farms rotation regimes focussed on annual crops of cereals and forage legumes; in some regimes forage legumes or *Sinapis arvensis*, mustard, were ploughed in as a green manure to enhance fertility.

**Table 1:** Information on the farms studied in Haute Provence

Producers emphasised the importance of growing cereal varieties adapted to stony soils of moderate to low nutrient status. These included a local variety of einkorn wheat, protected under an IGP (Indication Géographique Protégée) designation. This variety was almost always autumn-sown (normally in October) and had the longest growing season, ripening in August or even September (as in 2013—a particularly wet summer—on the plateau d’Albion) after the other cereals and lavender had been harvested. Another local variety was the blé meunier d’Apt, a low-gluten bread wheat (*Triticum aestivum*) revived in the 1980s in the northern Lubéron and also protected as an IGP landrace. Other (commercial) varieties included several other naked wheats (mostly hexaploid), *T. spelta*, spelt (on one plateau d’Albion farm), six-rowed hulled barley (*Hordeum vulgare*), rye (*Secale cereale*) and oats (*Avena* spp.) Cereals were mostly autumn-sown; spring-sown cereals followed autumn-sown ones in some rotation regimes. Pulses (*Lens culinaris*, lentil, and *Cicer arietinum*, chickpea) were sown over the winter or spring on some farms, in one case (Farm 2) only on stone-free alluvial soils.

Prior to sowing, fields were tilled by tractor, often using a disc plough. Though no hand-weeding of cereals is practised nowadays, cereals were harrowed in April around Sault and Saint-Christol in order to remove early spring weeds, with 2–3 passes in the case of einkorn, which was considered to recover better than other cereals due to its strong tillering capacity. Further south, on the more clay-rich soils of the northern Lubéron, harrowing was not practised since it tore up the crop due to the formation of a hard clay ‘crust’ on the soil surface. In one farm near Sault (Farm 2, Table 1), pulse fields were intensively worked by *vibroculteur* (a sort of harrow with vibrating teeth) to remove spring weed growth.

A broad contrast can be drawn between the clay-rich, calcareous soils of the northern Lubéron and the more friable soils around Sault that permitted spring harrowing but also tended to be acidic due to the formation of sesquioxides, and had in some areas been treated with lime in the past to raise the soil pH. One farmer (Farm 5) reported pH levels of c. 4.0 on his plateau d’Albion fields. Farmers also observed more local variation in soil type, ranging in the Sault area from loamy alluvial soils along the river Nesque, where soils were less stony and more humid, to fields on the plateau d’Albion covered by a continuous scatter of flint.

All of the farms surveyed were organic, being managed without chemical fertilisers or herbicides, and most had been so for decades (Table 1). Four of the seven farms practised no manuring with animal dung, relying entirely on crop rotation and in some cases a ‘green manure’ crop (forage legume or mustard) to maintain a modest level of fertility. Several farmers observed that cereals clearly benefited from the additional nitrogen fixed by a preceding leguminous crop, especially when ploughed in as a green manure. One farmer (Farm 6) also noted that ploughing in the bottom c. 20 cm of einkorn straw helped to maintain fertility, while another (Farm 2) had begun to spread rotted lavender straw (composted for 2 years) on fields to be planted with bread wheat or barley. On one Sault farm (Farm 3), the producer’s aunt raised sheep that provided dung for aromatic crops; sheep manure was applied at a rate of c. 30 t/ha in the fifth or sixth year of lavender growth, meaning that a hectare of land would receive c. 30 tons every 15 years or so. On the two farms (Farms 6–7) on alkaline soils in the northern Lubéron, fowl (and in one case also sheep) dung was applied to cereals at low rates (≤3 t/ha); one farmer indicated that fowl dung was applied in order to lower soil pH and hence to prevent the formation of calcium crystals on cereal roots that impede water uptake. Several farmers commented that sheep dung used to be applied to cereal fields since many farms formerly raised sheep alongside arable crops. A farmer in the northern Lubéron (Farm 1) observed that in the mid-20th century sheep were allowed to graze vegetative cereals on clay-rich soils, both in order to trample down roots raised by frosts and to prevent lodging (cf. Halstead 2006).

Cereals and pulses were harvested earlier in the northern Lubéron than in the Sault area; the timing varied from late June to August (September) depending on the crop and local setting, einkorn being harvested later (August or even September) than other cereals (Table 1). The 2013 harvest was about 15 days later than usual due to an abnormally wet and cool spring and summer.

Comparison of the cereal production regime in Haute Provence with a more intensive system in Asturias, Spain (Fig. 1) (Charles et al. 2002) using a combination of weed ecological and crop isotopic analysis is central to this study. Table 2 summarises climatic and agronomic contrasts between these two areas. Though both areas fall largely within the Cfb zone, Asturias experiences somewhat higher annual rainfall. Obvious agronomic contrasts between the Haute Provence and Asturias production regimes concern their scale and their intensity of management (Table 2).

**Table 2:** Comparison of Haute Provence and Asturias study areas

	Haute Provence	Asturias
Köppen–Geiger climate zone	Temperate-oceanic (Cfb) to temperate-mediterranean (Csb)	Temperate-oceanic (Cfb)
Annual precipitation (mm)	700–900	1,000–1,100
Farming scale	1–2 ha (<100 ha farms) to 8–9 ha fields (>100 ha farms)	Plots of 15–800 m^2^, few fields per producer
Manuring	Minimal (nil to few t/ha/year)	15–40 t/ha/year
Weeding	No hand-weeding; spring harrowing around Sault	Ploughing combined with hoeing to prepare soil; hand-weeding during growing season

## Materials and methods

### Field methods

In June–July, 2013, a floristic survey was carried out of the weeds in 57 cereal or pulse fields in this region, using methods comparable to those employed in similar surveys of the weeds associated with different husbandry regimes in Spain (Jones et al. 1995; Charles et al. 2002), Jordan (Palmer 1998; Charles and Hoppé 2003) and Greece (Jones et al. 1999). The weed species present in each of ten 1 m^2^ quadrats were recorded along a linear transect from one end of the field to the other. Three fields had distinct topographical subdivisions and were recorded using two separate transects, for a total of 60 field transects.

GPS data for individual fields and isotope samples were recorded using a handheld GPS unit during the field survey. GPS data recorded included the boundary of every field surveyed and the specific location of every quadrat within each field. The data were analysed using ArcGIS 10.1 in order to detect any associations between topographical variables and weed floristic composition or crop isotope values.

Ecological attributes of the most commonly occurring weed species identified in this survey were measured during the same study season. Species were selected for the measurement of functional attributes if they occurred in 5 % or more of the fields (i.e. three or more fields). These species are listed in Supplementary Table 1. The attributes measured were those found most useful for the identification of cultivation intensity in the original Evvia study (Jones et al. 2000), and are summarised in Table 3 (see Charles et al. 1997; Bogaard et al. 1999; Jones et al. 2000; Bogaard et al. 2001; Charles et al. 2003 for fuller explanations). Amphistomaty (relative distribution of stomata on upper and lower leaf surface), an indicator of shade tolerance, was excluded here since stomatal attributes were not measured in the Provence study.

**Table 3:** The weed functional attributes measured and their possible ecological significance within an arable context

Functional attribute measured	Ecological attribute for which measurement is a surrogate	Relationship to habitat conditions	References
Attributes relating to the duration and quality of the growth period			
Maximum canopy height and diameter	Maximum plant size, the product of growth rate and period of growth	Positively correlated with potential productivity, negatively with disturbance of habitat	Grime (1979), Moles et al. (2009)
Leaf area per node/leaf thickness	Plant growth rate	Positively correlated with potential productivity of habitat	Jackson (1967), Dale (1982), Givnish (1987), Jones et al. (2000)
Mean specific leaf area (leaf area/dry leaf weight)	Plant growth rate	Positively correlated with potential productivity of habitat	Reich et al. (1992), Reich (1993), Wright et al. (2004), Poorter et al. (2009)
Attribute relating to seasonality and/or the capacity to regenerate under conditions of high disturbance			
Length of flowering period	Duration of life cycle and potential to regenerate from seed	Positively associated with disturbance	Grime et al. (1988), Sans and Masalles (1995), Bogaard et al. (2001)

Only well-grown specimens were selected for measurement of functional attributes so that *species* potential was assessed rather than individual plant performance under variable conditions. Detailed protocols for the measurement of each of these attributes are given in Jones et al. (2000) and Bogaard et al. (2001). Canopy height and diameter were converted to a log scale. Attributes such as duration and onset of flowering were extracted from Rothmaler (1995).

A total of 23 pairs of isotopic measurements (δ^13^C and δ^15^N) was obtained from 24 fields in Haute Provence (two, ABI01 and ABI02, were merged); those fields to which fowl dung had been applied (Table 1) were excluded due to the distinctive effect of this form of manure on plant δ^15^N values (cf. Szpak 2014). In some cases crops were collected ripe during the weed surveys. In that case, one ear was taken from each of the ten quadrats. For fields where ripe crops were obtained after the harvest, ten random ears were chosen across each field. Grains from these ears were threshed by hand, and a random subset of 50 was powdered using a Spex 2760 FreezerMill. Since inter- and intra- ear isotopic variability can be significant (Bogaard et al. 2007; Heaton et al. 2009) homogenizing a large (n > 15) number of seeds ensures that measurements are adequately representative of the average signature for each field (Kanstrup et al. 2012; Nitsch et al. submitted).

### Laboratory analysis

The homogenized powders were weighed into tin capsules for IRMS analysis on a SerCon EA-GSL mass spectrometer, with δ^13^C and δ^15^N measured separately. An internal alanine standard was used to calculate raw isotopic ratios. For δ^13^C two-point normalization to the VPDB scale was obtained using four replicates each of IAEA-C6 and IAEA-C7, while for δ^15^N the standards were USGS40 and IAEA-N2. Reported measurement uncertainties are the calculated combined uncertainty of the raw measurement and reference standards, after Kragten (1994). The average measurement uncertainty for δ^13^C was ±0.07 and ±0.35 ‰ for δ^15^N. These calculations were performed using the statistical programming language R. (3.0.2).

### Data analysis

In the modern weed studies discussed here, the number of quadrats out of ten in which each taxon occurred was recorded. By contrast, archaeobotanical data, to which the model developed here is ultimately applied, are quantified on the basis of numbers of seeds. The quantitative data from the modern weed survey studies are therefore not directly comparable with archaeobotanical data. Analysis was therefore conducted in two ways: quantitatively (using the number of quadrats in which each taxon was found); and semi-quantitatively (using presence/absence of species in each cultivated field). The latter is more directly comparable between modern and archaeobotanical datasets but involves some loss of information.

Discriminant analysis was used to distinguish crop fields cultivated under the two different intensity regimes, in Haute Provence and Asturias. For this purpose, an average score for each attribute was determined for each cultivated field as follows:

$${{\sum \!{_{i}^{n} a_{i} k_{i} } } \mathord{\left/ {\vphantom {{\sum {_{i}^{n} a_{i} k_{i} } } {\sum {_{i}^{n} k_{i} } }}} \right. \kern-0pt} {\sum \!{_{i}^{n} k_{i} } }}$$

For the quantitative measure: k_i_ is the no. of quadrats in which the ith species was recorded, a_i_ is the value of attribute for the ith species, and n is the no. of species recorded in each field.

For the semi-quantitative measure, k is always equal to 1 and so the numerator is simply the sum of the attribute values for the species in the cultivated field and the denominator is the number of species in each field.

The success of the discriminations was measured in terms of the percentage of fields correctly reclassified as ‘low intensity’ (in Haute Provence) or ‘high intensity’ (in Asturias), using the discriminant function extracted in each analysis. The same discriminant functions were used to classify the cereal fields from Sighisoara and Kastamonu and archaeobotanical samples from Neolithic central Europe, all of which were entered into the classification phase of the analysis as samples of ‘unknown’ cultivation regime. SPSS version 20.0 was used to perform discriminant analyses, using the ‘leave-one-out’ option.

Canoco for Windows 4.5 and CanoDraw for Windows (ter Braak and Smilauer 2002) were used to carry out correspondence analyses of the weed survey data from Haute Provence in order to explore variation in floristic composition among fields. In the figures, correspondence axis 1 is plotted horizontally and axis 2 vertically. Statistical calculations related to the isotopic data were performed using the statistical programming language R. (3.0.2). Comparisons between the four different study regions were calculated using a one-way ANOVA, with pairwise differences reported as 95 % confidence intervals with Tukey-HSD-adjusted *p* values.

For the Haute Provence study, topographic analysis was based on the BDALT25 digital elevation model (URL: http://professionnels.ign.fr/bdalti#tab-1). A wide range of topographic variables was analysed, using the Hydrology, Solar Radiation and Surface toolsets in ArcGIS 10.1.

## Results

### Causes of floristic variation in the Haute Provence weed flora

**Fig. 3:**
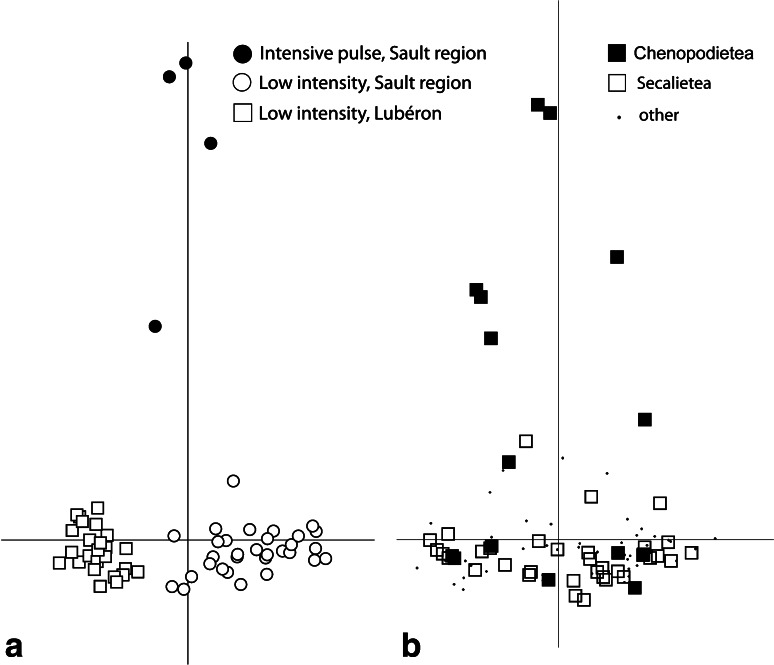
Correspondence analysis of Haute Provence fields and weed taxa: **a** plot of 60 field transects, coded by regime and region; **b** plot of weed species, coded by phytosociological class (Chenopodietea = root/row-crop ‘garden’ weeds; Secalietea = cereal field weeds). *Axis 1* horizontal, *axis 2* vertical

Correspondence analysis of all 60 crop field transects in Haute Provence (Fig. 3a) on the basis of 78 weed species occurring in at least 5 % of fields presents a clear contrast on axis 1 (horizontal) between the Sault region (right) and the Lubéron (left). Axis 2 (vertical) distinguishes between the majority of fields (including both cereals and pulses) concentrated at the negative (bottom) end from four intensively worked pulse fields towards the positive end. These were fields of spring-sown lentils and chickpeas on loamy stone-free soils along the river Nesque near Sault (Farm 2, Table 1) ‘weeded’ mechanically using a *vibroculteur* once the early spring weeds had germinated. The weed species associated with the intensively worked pulse fields belonged in phytosociological terms to the Chenopodietea, garden or row-crop weeds (Fig. 3b). Although some Chenopodietea species are associated with low intensity cereal (and pulse) cultivation, the vast majority of species associated with low intensity cultivation belong to the class Secalinetea. The small number of fields did not permit full ecological characterisation of the more intensive form of pulse cultivation in Haute Provence, but the evident contrast with less intensively managed fields is relevant to comparison with intensive cultivation in Asturias (see below).

**Fig. 4:**
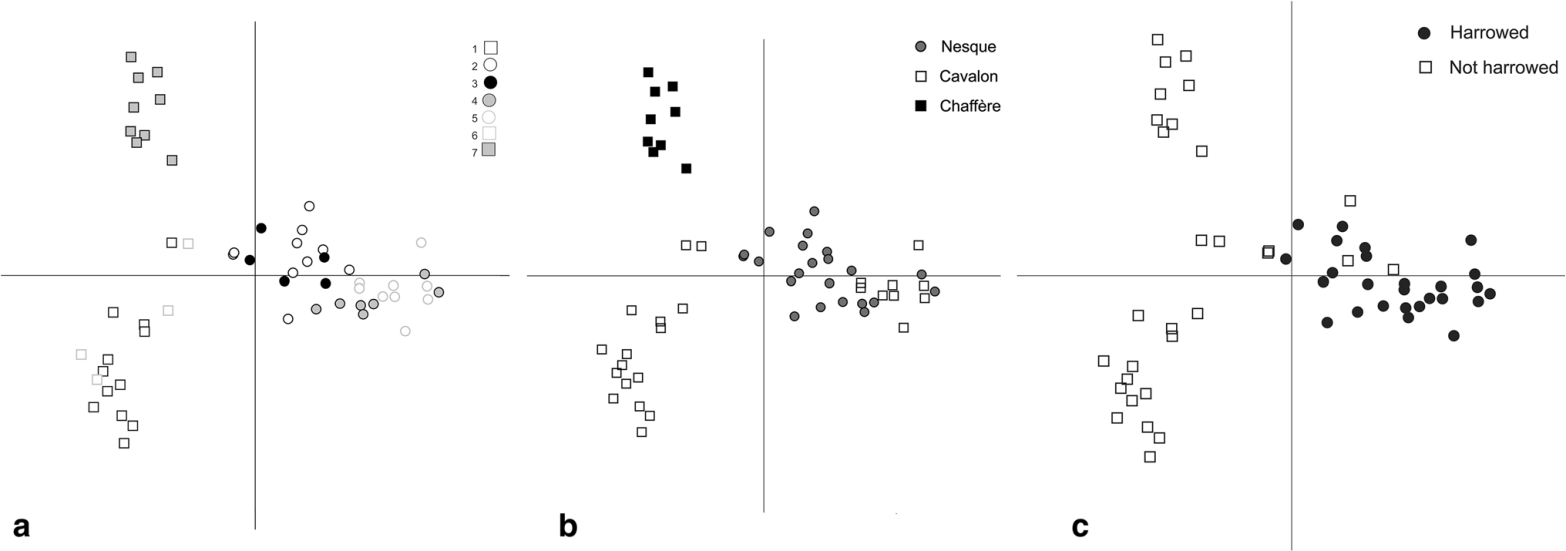
Correspondence analysis of the weed species in 56 Haute Provence field transects (excluding intensively cultivated pulses), plot of transects coded by **a** farm (see Table 1); **b** river drainage (see Fig. 2); **c** harrowing. *Axis 1* horizontal, *axis 2* vertical; *circles* Sault area, *squares* Lubéron area

In a second correspondence analysis, excluding the intensively worked pulse fields, the geographical distinction between the Sault area (right) and the Lubéron (left) remains dominant on axis 1 (Fig. 4a). Axis 2 distinguishes the Cavalon and Chaffère drainages in the Lubéron (Fig. 4b). Figure 4a, coded by farm, reveals subregional clustering of farms in the Sault region and Cavalon drainage; the Chaffère drainage (upper left) is represented by a single farm. The dominance of geography as a correlate with floristic variation in Haute Provence indicates a contrast with the study of ‘garden’ and ‘field’ cultivation in Evvia, Greece, where any geographical variation in the weed flora was subordinate to the gradient in agricultural intensity (Jones et al. 1999).

**Fig. 5:**
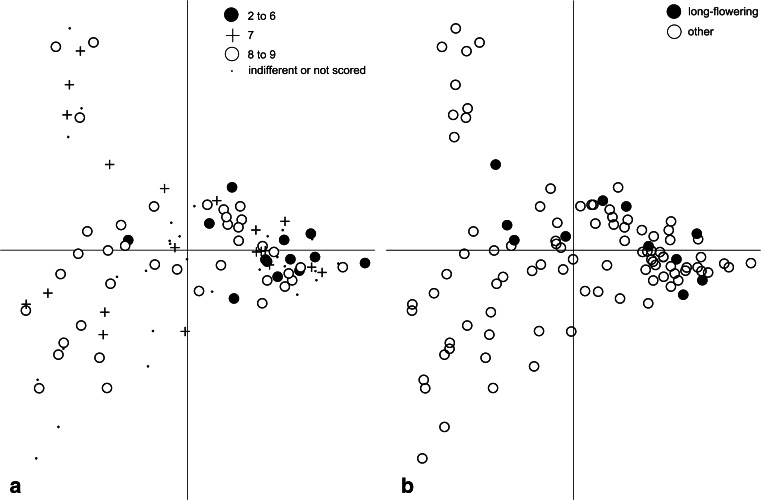
Correspondence analysis of the weed species in 56 Haute Provence field transects (excluding intensively cultivated pulses), with plot of species coded by **a** Ellenberg reaction (pH) scale (a 1–9 scale where *1* indicator of extreme acidity, *7* weakly acidic to weakly basic and *9* basic reaction and lime indicator, always found on calcareous soils—Ellenberg et al. 1992); **b** flowering duration. *Axis 1* horizontal, *axis 2* vertical

While the contrast between the Cfb climate of the Sault region and the more mediterranean climate of the Lubéron likely contributes to the geographical trend on axis 1, the contrast also involves different soil types. This is reflected in a contrast in weed species between those preferring acidic soils around Sault and those preferring alkaline soils in the Lubéron (Fig. 5a). These different soil types also encourage different agricultural practices: spring harrowing of cereals is associated with the soil types of the Sault/Albion plateau but is not practiced on the more clay-rich soils in the Lubéron (Fig. 4c). This explains the tendency for long-flowering annuals, able to recover from disturbance, to be located towards the positive (right) end of axis 1 (Fig. 5b). It is clear, however, that it is the location of fields (which determines soil type), rather than harrowing, that governs the distribution of fields in the correspondence analysis, because the few unharrowed fields in the Sault area cluster with the other (harrowed) Sault fields rather than the unharrowed fields of the Lubéron (Fig. 4), and the association of weed species preferring acidic soils with the Sault area is stronger than the association of long-flowering species with harrowing (Fig. 5).

As noted above, manuring of cereals in the Lubéron with chicken dung is a means of reducing the soil pH; manuring rates are very low, however, and no differences in weed functional attributes relating to soil productivity are apparent (plots not shown). Finally, neither crop sowing time nor crop taxon show any clear patterning (plots not shown). For example, the analysis did not detect any differences in the weed flora of einkorn fields due to their later harvesting time.

Overall, the weed flora of the Provence fields primarily vary according to their geographical location (in response to soil type and perhaps climate), and only secondarily in response to agricultural practice (notably harrowing).

### Ecological comparison of Haute Provence and Asturias regimes on the basis of weed functional attribute values

Discriminant analysis was used to distinguish between the weed flora of large-scale/low-intensity production in Haute Provence and small-scale/intensive cereal farming in Asturias, on the basis of the five functional attributes previously shown to distinguish between high- and low-intensity pulse cultivation regimes in Evvia, Greece as discriminating variables (Table 3). Though geographical differences between the two areas may affect the discrimination (Jones et al. 2010), our hypothesis was that the strong agronomic contrasts (Table 2) would enable the regimes to be distinguished along predictable lines using these attributes. All of the Asturias fields surveyed (Charles et al. 2002), and 56 of the Haute Provence field transects, were included in the discrimination; the four intensively worked pulse fields in Haute Provence (Fig. 3a) were entered into the classification phase of the discriminant analysis.

**Fig. 6:**
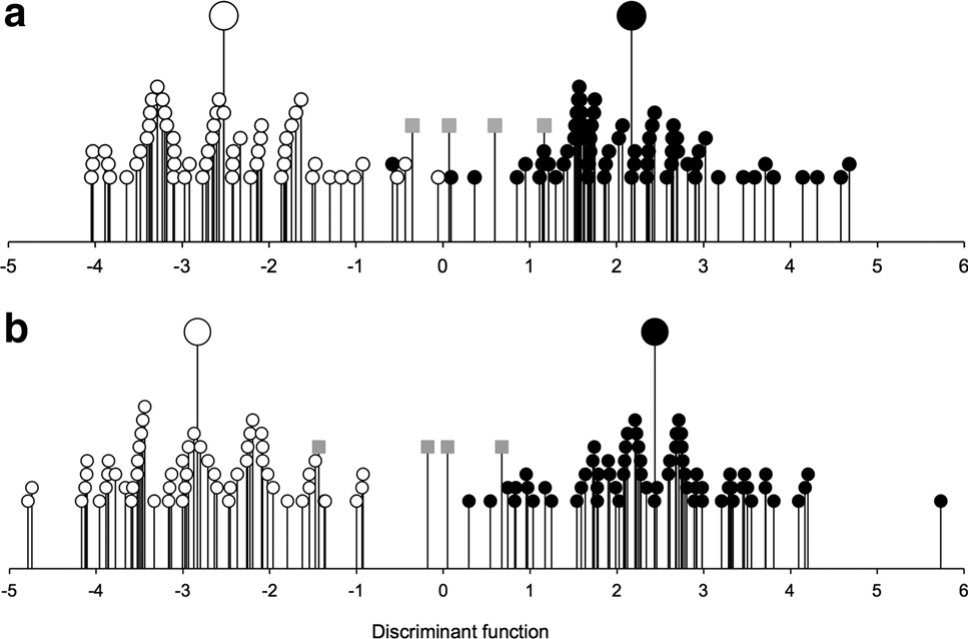
The relationship of Haute Provence fields (*open circles*, n = 56) and Asturias fields (*filled circles*, n = 65) to the discriminant function extracted to distinguish these two groups on the basis of **a** fully quantitative; **b** semi-quantitative (presence/absence) weed attribute scores. *Larger symbols* indicate group centroids; *grey squares* intensively worked pulse fields in Haute Provence entered into the classification phase of the analysis only

**Fig. 7:**
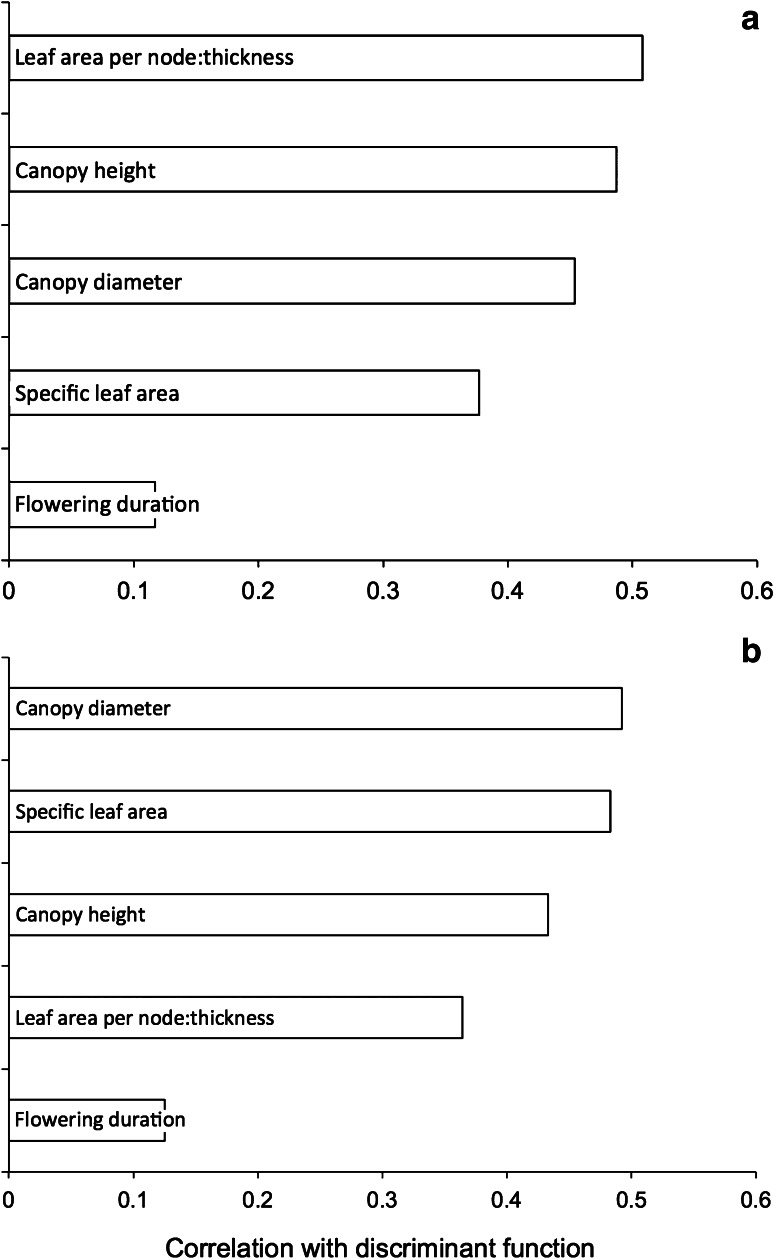
Correlations between the functional attribute scores used as discriminating variables and the discriminant function based on **a** fully quantitative data; **b** semi-quantitative (presence/absence) data

Figure 6 shows that the Haute Provence and Asturias fields are clearly distinguished on the basis of fully quantitative (98 % correctly reclassified, 119 of 121) and semi-quantitative (presence/absence) data (100 % correctly reclassified; Fig. 6). Functional attributes characterise the two regimes in accordance with ecological predictions (Fig. 7). In both analyses, weed species with tall, broad canopies, high specific leaf area (SLA) and a high leaf area:thickness ratio are associated with intensive cereal farming in Asturias, and vice versa for the less fertile Provence fields; a long flowering period is also associated with greater disturbance in Asturias (cf. Jones et al. 2000). It is worth noting that three of the four more intensively worked pulse fields in Haute Provence—those that emerged as distinct in the correspondence analysis (Fig. 3a)—were classified as intensively cultivated by both discriminant functions (Fig. 6).

The functional attributes discriminate between the Haute Provence and Asturias regimes as predicted based on contrasts in agronomic practice, despite geographical differences between the two areas (Table 2; cf. Jones et al. 2010). Moreover, pronounced geographic variation within Haute Provence (above) does not obscure the predicted ecological contrast with Asturias.

### Comparison of Haute Provence and Asturias regimes on the basis of cereal stable isotope values

**Fig. 8:**
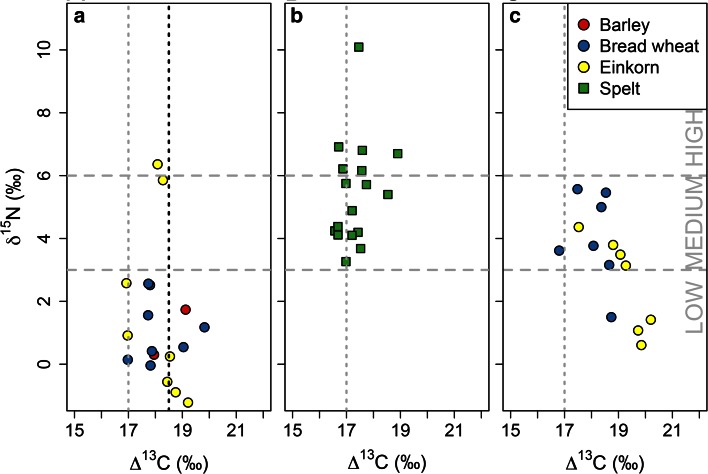
A scatter plot of Δ^13^C versus δ^15^N values for modern cereal samples collected from **a** Haute Provence, France, **b** Asturias, Spain, and **c** Sighisoara, Romania. *Vertical lines* indicate reference values for well-watered wheat (*grey*) and barley (*black*) (after Wallace et al. 2013). *Horizontal lines* indicate reference values for high, medium and low levels of manure (after Bogaard et al. 2013)

Table 4 and Fig. 8 show that the production regimes in Haute Provence and Asturias exhibit largely distinct ranges of δ^15^N values (a difference of 2.36 ‰, 95 % CI 0.71, 4.01 ‰, *p* = 0.002). The Asturias fields exhibit a range of values equivalent to ‘medium’ and ‘high’ levels of manure application in long-term agricultural experiments (Fraser et al. 2011). Agronomic observations in Asturias show that manure is applied at rates equivalent to c. 15–40 tons per year for 1 ha (Table 2; Bogaard 2012), spanning experimental medium (c. 10–15 t/ha) to high (30+ t/ha) rates (Fraser et al. 2011). With two exceptions, the Haute Provence fields exhibit δ^15^N values lower than 3 ‰, as predicted by long-term agricultural experiments where there is continuous cultivation with little to no manuring (Fraser et al. 2011). The exceptions are two einkorn fields (Farm 3, Table 1) located near Sault along the river Nesque. Manuring can be excluded as a cause of the high δ^15^N values for these two fields, which were managed in the same way as the other fields on Farm 3), so natural organic matter or anaerobic denitrification are more likely explanations (see also below).

**Table 4:** Mean crop stable isotope values and discriminant scores for the weed functional attribute analysis of the modern cereal studies

	Mean	δ^15^N		Mean	∆^13^C		Discriminant scores				
							Fully quantitative		Semi-quantitative		
		SD	N		SD	N	Mean	SD	Mean	SD	N
Haute Provence	1.2	2.2	19	18.1	0.8	19	−2.5	1.0	−2.8	1.0	56
Asturias	5.4	1.7	17	17.3	0.6	18	2.2	1.0	2.4	1.0	65
Sighisoara	3.3	1.6	14	18.6	1.0	14	nd	–	1.8	1.5	17
Kastamonu	1.3	1.7	8	nd	–	–	nd	–	−3.3	1.9	13

Higher δ^15^N values coincide with higher mean discriminant scores in the weed functional attribute analysis for Asturias, and vice versa for Haute Provence (Table 4). This relationship reflects the contribution of manuring to the high cultivation intensity ‘signature’ of the weed species in Asturias.

Carbon discrimination (∆^13^C) values in Asturias and Haute Provence suggest that cereals in both regions generally had access to sufficient water (∆^13^C > 17 ‰ for wheat, Wallace et al. 2013). Values in Haute Provence tend to be higher (1.24 ‰, 95 % CI 0.67, 1.82 ‰, *p* < 0.001) despite lower annual rainfall than those from Asturias (Table 2), potentially due to an exceptionally wet spring/summer when the Haute Provence study was conducted in 2013, and/or the [stress-tolerant] crop varieties grown in Haute Provence.

### Comparison with ‘traditional’ cereal production regimes in Romania and northern Turkey

**Fig. 9:**
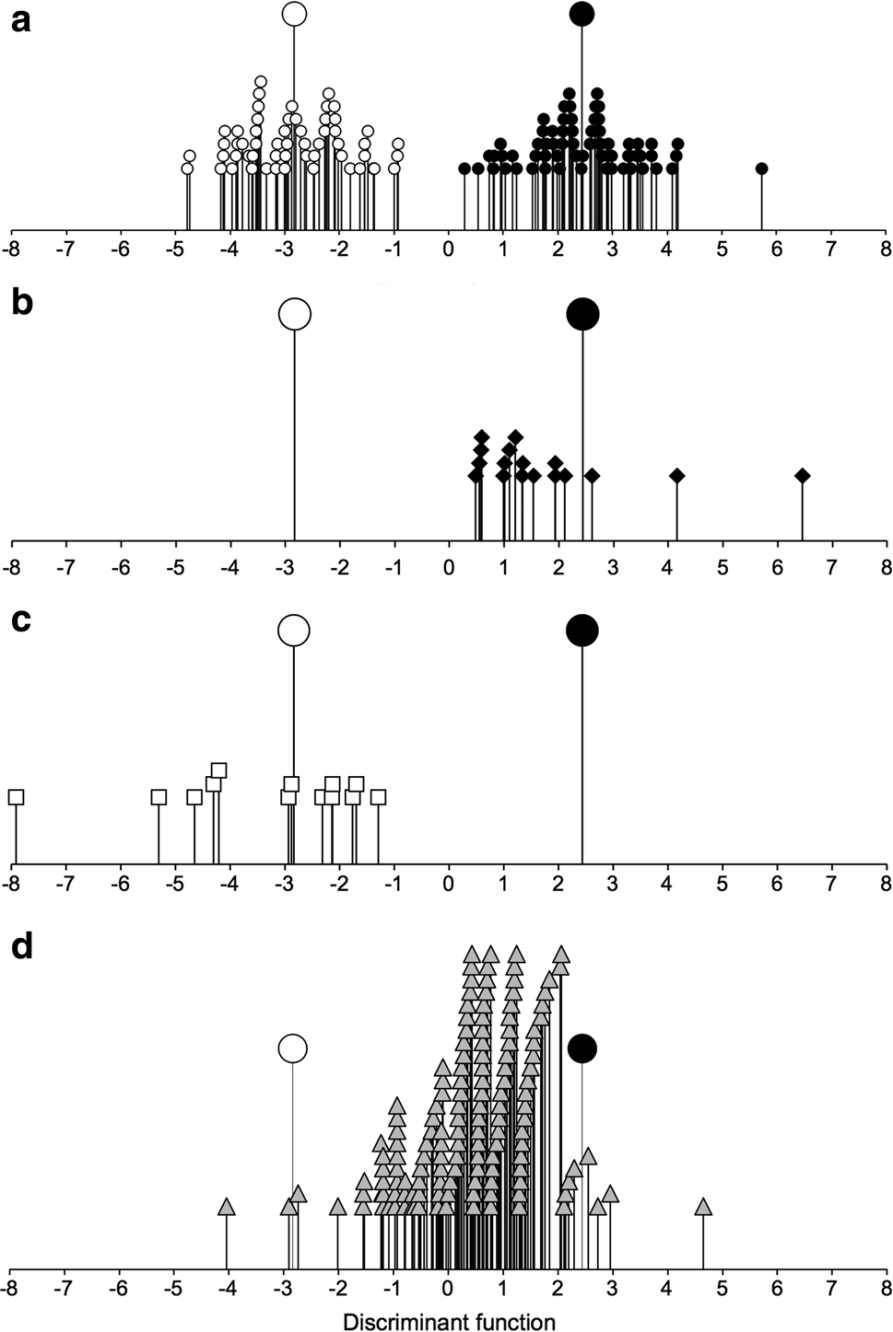
The relationship of **a** Haute Provence fields (*open circles*, n = 56) and Asturias fields (*filled circles*, n = 65), **b** Sighisoara fields (*filled diamonds*, n = 17), **c** Kastamonu fields (*open squares*, n = 13) and **d** archaeobotanical samples from Neolithic central Europe (*grey triangles*, n = 141) to the discriminant function extracted to distinguish the Haute Provence and Asturias groups on the basis of semi-quantitative (presence/absence) weed attribute scores. *Larger symbols* indicate centroids for Haute Provence and Asturias

In order to test the accuracy of the new ‘weed plus isotope’ intensity model, we first used the discriminant function extracted to separate the Haute Provence and Asturias fields on the basis of semi-quantitative data (i.e. the form in which it would be applied archaeobotanically) to classify intensively managed crop fields in a study area in Transylvania, near Sighisoara, Romania (Fig. 1), in a region of temperate-continental climate (Köppen–Geiger zone Dfb). Here, einkorn and other cereals are grown at c. 350–600 m altitude in small fields (c. 50–1,500 m^2^) with variable levels of manuring (often applied to maize or potatoes, grown in rotation with the cereals) and hand-weeding (Hajnalová and Dreslerová 2010; Fraser et al. 2011). The weeds in a total of 17 cereal fields in this region were surveyed in 2008 using the same methods as in Haute Provence and Asturias; 14 fields with ripe crops (including eight surveyed for weeds) were sampled for crop stable isotope analysis. Table 5 and Fig. 9 show that the Sighisoara fields were all (correctly) classified as ‘intensive’. It is notable that most discriminant scores for the Sighisoara fields are located at the ‘low-intensity’ end of the Asturias spectrum; this result corresponds well with agronomic observations, which indicate that the Sighisoara fields were managed rather less intensively than most of the Asturias fields [e.g. less consistent manuring and hand-weeding: see Charles et al. (2002), Hajnalová and Dreslerová (2010)].

**Table 5:** Summary of the classification of Sighisoara and Kastamonu fields by the discriminant function extracted to distinguish the Haute Provence and Asturias regimes on the basis of weed functional attributes

p*	High-intensity (Asturias)	Low-intensity (Haute Provence)	Total
Sighisoara fields			
High	17	–	17
Low	–	–	
Total	17	–	17
Kastamonu fields			
High	–	13	13
Low	–	–	–
Total	–	13	13
Archaeobotanical samples			
High	83	30	113
Low	19	9	28
Total	102	39	141

* Probability of classifications: ‘high’ = ≥ 0.9, ‘low’ = < 0.9

**Fig. 10:**
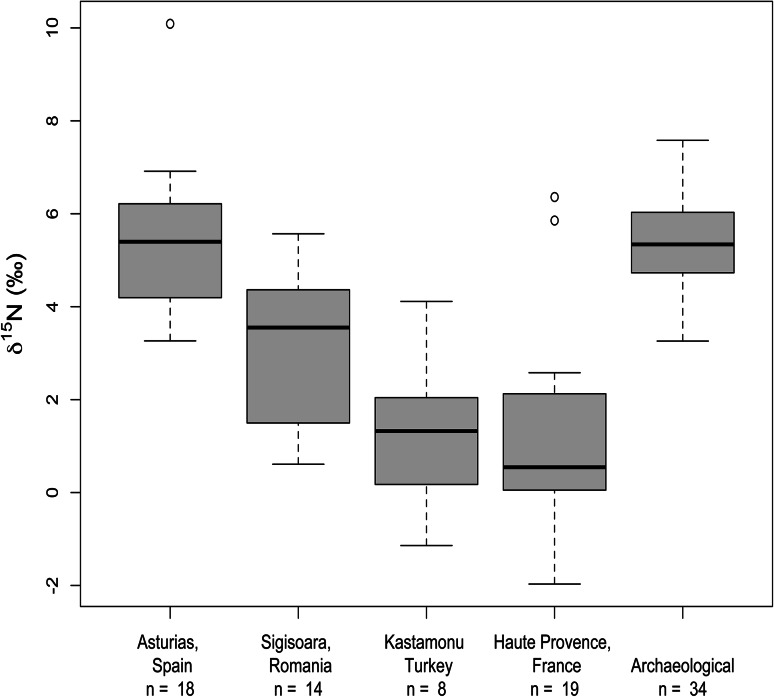
Box plots showing δ^15^N differences between modern cereals grown in four different locations and archaeobotanical cereals from Neolithic central Europe. The charred archaeobotanical specimens have been corrected for the effect of charring by subtracting 0.31 ‰ after Nitsch et al. (2015)

Next we compared the Sighisoara fields with those in Asturias and Haute Provence in terms of their crop isotope values (Table 4; Figs. 8c, 10). The majority of Sighisoara fields (11 of 14) have δ^15^N values consistent with ‘medium’ rates of manuring (Fraser et al. 2011; Bogaard et al. 2013), while a small number have lower values and had not been manured in recent years (only one of the latter was included in the weed surveys). In terms of ∆^13^C values, those of the Sighisoara einkorn tend to be higher (‘wetter’) than those of bread wheat by an average of 1.1 ‰ (Fig. 8c; t(12) = 2.60, *p* = 0.023). Since the einkorn fields were managed in the same way as the bread wheat crops, and on similar soils, it appears that the relationship between carbon discrimination and water status is different in Romanian einkorn and bread wheat; a similar ‘offset’ has previously been noted between barley and free-threshing wheat (Araus et al. 1997; Wallace et al. 2013). Such an offset is not apparent between einkorn and other wheats in Haute Provence (Fig. 8a), and may reflect landrace-specific variation.

In order to test the ability of the new model to correctly identify *low*-intensity cereal production, we applied it to a second case study area, centred around the town of İhsangazi in the (Cfb) Kastamonu province of northern Turkey (Fig. 1), where einkorn and emmer were grown on a moderately large scale (fields ranging from c. 0.2 ha to 4.5 ha) with little to no manuring and no weeding, in rotation regimes incorporating forage legumes and bare (ploughed) fallow (Karagöz 1996; Ertuğ^2004^). The weeds in a total of 13 cereal fields in this region were surveyed in 2008 using the same methods as in Haute Provence and Asturias; eight fields with ripe crops (including seven surveyed for weeds) were sampled for crop stable isotope analysis. Table 5 and Fig. 9 show that the Kastamonu fields are all classified correctly, on the basis of their weed functional attributes, as being subject to low-intensity cultivation, in one case with a much lower discriminant score than the Haute Provence fields, suggesting poorer growing conditions than in the Haute Provence study.

We then compared the Kastamonu fields with those in the other studies in terms of their δ^15^N values (see Fraser et al. 2011 for the isotope methodology pertaining to these samples); ∆^13^C values are not available for these samples (Table 4; Fig. 10). Seven of the eight Kastamonu fields have δ^15^N values below 3 ‰, consistent with their reported history of little to no manuring; the cause(s) of the single higher value (4.1 ‰), from a field not manured in recent years, is unclear.

### The relationship between weed ecology and cereal isotope values on a field-by-field basis

**Fig. 11:**
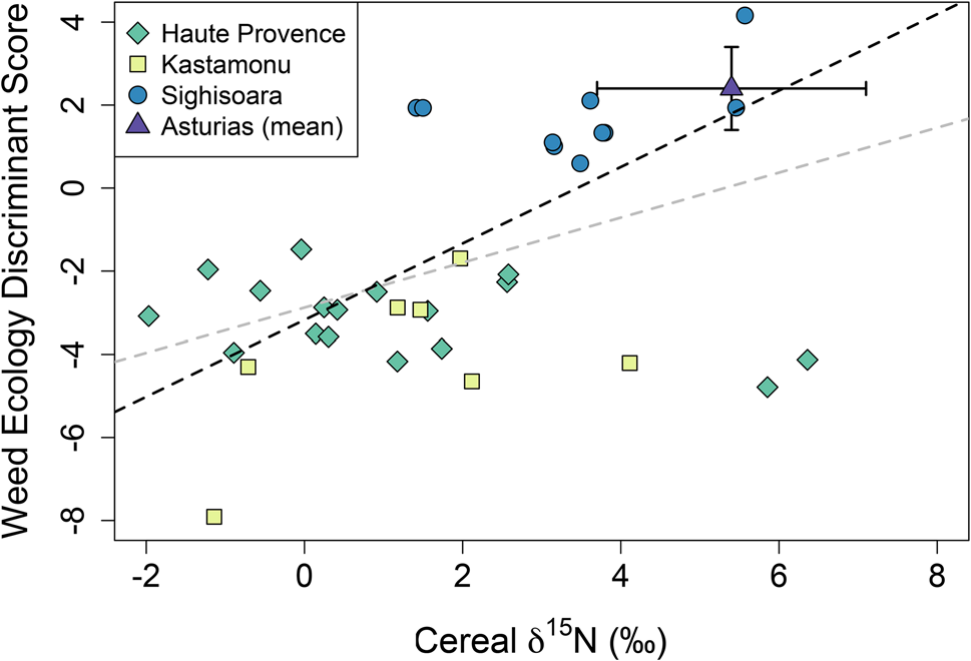
A scatter plot of the weed ecology discriminant scores versus δ^15^N values for modern cereals grown in four different locations, including the mean and 1σ range of the discriminant scores and δ^15^N values for Asturias. The *grey* linear regression line (y = 0.54x−2.88) is based on all plotted data (R^2^ = 0.19), the *black* linear regression line (y = 0.92x−3.18) excludes two outlying transects from Provence with high δ^15^N values (R^2^ = 0.45)

Figure 11 shows the relationship between discriminant scores based on (semi-quantitative) weed ecological data and δ^15^N values for cereals in the Haute Provence, Sighisoara and Kastamonu studies (n = 34), where the two variables can be compared on a field-by-field basis; there is a weak but significant positive correlation for all fields (R^2^ = 0.19, *p* = 0.009). This is consistent with the agronomic observation that manuring contributes to the contrast in intensity between Sighisoara, on the one hand, and Haute Provence and Kastamonu, on the other. Field discriminant scores and isotope values for the same fields are not available for Asturias, but Fig. 11 includes the mean ±1σ for all Asturias weed survey transects and cereal isotope samples. The Asturias data fit well within the overall pattern.

The two einkorn fields from Provence (Farm 3) with unusually high cereal δ^15^N values emerge as outliers in Fig. 11; they do not have a high cultivation intensity signature based on their weed functional ecology. In fact, their weed ecological signature places them at the ‘low-intensity’ end of the Provence range. We hypothesise that these elevated δ^15^N values are due to seasonal waterlogging along the river, rather than to high organic matter content (through manuring or natural abundance). This example illustrates the value of combining crop isotope analysis with weed ecology: interpretation of high crop δ^15^N values is informed by ecological analysis of the associated weed flora. If these two fields are excluded, the correlation between the weed ecology discriminant scores and cereal δ^15^N values is stronger (R^2^ = 0.45, *p* < 0.001).

**Fig. 12:**
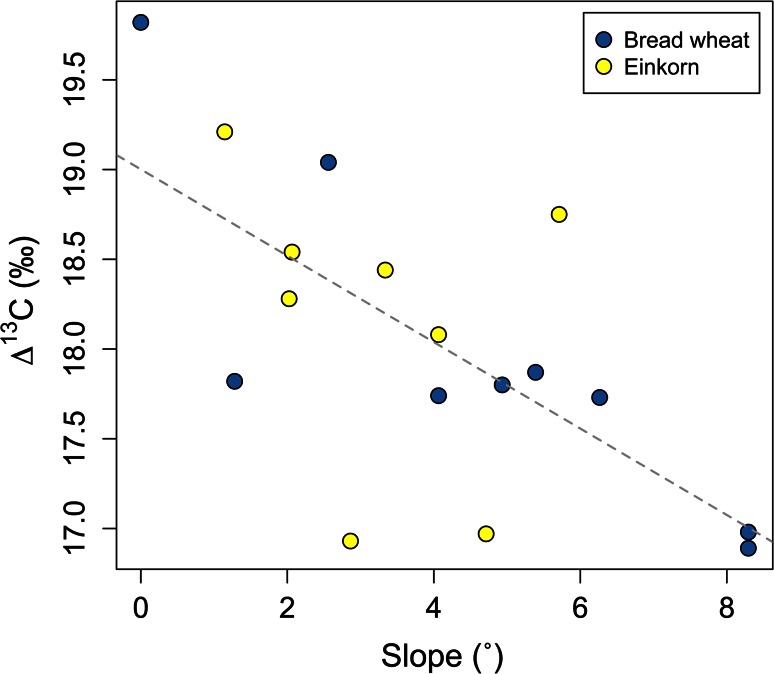
A scatter plot of degrees of field slope versus Δ^13^C values of cereals in Haute Provence, showing linear regression line (y = 38.6x−1.9, *p* = 0.0011, R^2^ = 0.47)

There was no correlation between cereal ∆^13^C values and weed ecology discriminant scores; this is to be expected since artificial watering did not form part of management intensity in these studies. Topographical analysis of the fields using GIS, however, did reveal a relationship between slope and cereal ∆^13^C values (Fig. 12), such that steeply sloping fields are associated with lower (‘drier’) values than flat fields (R^2^ = 0.45, *p* = 0.003). This relationship presumably reflects greater water run-off on slopes. No other topographical variables (aspect, solar radiation, distance from stream/river) exhibited a clear relationship with cereal ∆^13^C or δ^15^N values.

### Application of the Asturias/Haute Provence model to archaeobotanical data from Neolithic central Europe

The discriminant function extracted to distinguish the high-intensity cultivation in Asturias and low-intensity cultivation in Haute Provence on the basis of semi-quantitative weed ecological data was used to classify 141 Neolithic archaeobotanical samples from 30 sites across central Europe (Supplementary Table 2). These samples were selected because each derives from a single ‘deposit’, contains a minimum of 30 potential weed seeds identified to species and contains crop and weed material consistent with a single crop processing stage; this set of samples includes the 126 discussed in Bogaard (2004), another 14 samples subsequently analysed from Vaihingen/Enz (Bogaard 2012) and a weed-rich pit fill from Ecsegfalva (Bogaard et al. 2007). Most of these samples belong to the early Neolithic *Linearbandkeramik* (LBK) complex; half come from Vaihingen (Supplementary Table 2) (Bogaard 2012).

Table 5 and Fig. 9 show that most samples (71 %, 100 out of 141) are grouped with the Asturias fields, and the remainder with Haute Provence transects. A significant proportion of the archaeobotanical samples (41 %, 57 out of 140) are classified with low (<0.90) probability, and are positioned between the two groups on the discriminant function in Fig. 9d. Most of the archaeobotanical samples appear to derive from conditions akin to the ‘low-intensity’ end of the Asturias spectrum, in a manner reminiscent of the Sighisoara fields. While a significant minority of samples are grouped with the Provence transects, most are placed at the ‘high-intensity’ end of the Haute Provence spectrum, with only a few samples around or below the Provence group centroid.

Overall, the archaeobotanical results suggest a range of agricultural conditions centred around the medium to high intensity part of the spectrum. While the problem-oriented approach of classifying archaeobotanical samples as unknown cases in discriminant analysis has a limited ability to detect regimes without a present-day analogue (Jones et al. 2010), it is evident that the vast majority of the archaeobotanical samples point to a consistent range of conditions between the Asturias and Haute Provence groups, suggesting a distinctive set of intermediate conditions. Variation in growing conditions is to be expected, particularly in small-scale subsistence production where labour is limiting and is invested strategically depending on its availability (Halstead 2014).

Previous application of the Evvia pulse intensive ‘garden’ versus extensive ‘field’ cultivation model to the weeds of Neolithic central European (Bogaard 2004, 2012) mostly identified the samples as deriving from intensive cultivation, with few samples classified as extensively cultivated. The Evvia model identified attributes relating to both soil fertility (e.g. specific leaf area) and disturbance (flowering period) as important to the discrimination between intensive and extensive cultivation (Jones et al. 2000; Charles et al. 2002, Fig. 3) whereas soil disturbance (flowering period) was less important in the discrimination between intensive cultivation in Asturias and low-intensity cultivation in Haute Provence. Nevertheless, a consistent outcome from application of both the Evvia and the Haute Provence/Asturias models has been that the Asturias fields appear on the whole more intensively managed than the regime(s) represented by the central European archaeobotanical samples (cf. Bogaard 2004, 110). Taking the classifications of the Neolithic samples by the Evvia and Asturias/Provence models together, it is plausible that the Neolithic cultivation plots tended to be more disturbed but less fertile than the Asturias plots.

Full application of the new ‘multi-stranded’ model requires measurement of crop δ^15^N and ∆^13^C values in a large number of archaeobotanical samples. At present, only a pilot study in archaeobotanical crop isotope determination has been undertaken from a few Neolithic sites in central Europe, including Vaihingen (Bogaard et al. 2013). Cereal δ^15^N values from this work are comparable with those from Asturias and Sighisoara (Fig. 10). Combined consideration of weed ecology and crop isotope based inferences on crop growing conditions at Neolithic sites in central Europe suggests that cultivation was of an intensive type but that management intensity was variable.

## Discussion and conclusions

The intensity of cultivation refers to the level of labour inputs per unit area of arable land, and its archaeological recognition is crucial for assessing the social and ecological context of past farming regimes. In temperate regions where soil nutrients rather than water limit crop productivity, manuring is one of a set of labour-intensive practices (alongside thorough tillage and hand-weeding, for example) that enhance crop yields. High labour intensity, however, limits the scale of production, and an alternative strategy is to increase *absolute* yields by expanding the scale of farming (‘extensification’), with a corresponding decrease in intensity and area yields. Conflicting views of agricultural development in prehistory hinge on the timing and extent of agricultural expansion (e.g., Halstead 1995, 2014; Sherratt 1997). Explicit archaeobotanical models of cultivation intensity, ideally incorporating multiple strands of evidence, are necessary for addressing this debate (Jones 2005), alongside complementary lines of enquiry into, for example, livestock management and the palaeoecology of arable landscapes.

Our results demonstrate that weed functional attributes previously identified to distinguish low- and high-intensity pulse cultivation in Evvia, Greece (Jones et al. 2000) can, indeed, be applied to comparison of low- and high-intensity cereal regimes, and in geographical areas of contrasting climate. Presence/absence data proved to be just as reliable as fully quantitative data for this identification, which is encouraging for the application of these attributes to archaeobotanical data where the basis of quantification (number of seeds per sample) is different.

Isotopic analysis of cereal grain samples from Haute Provence and Asturias have shown that δ^15^N values mostly vary in accordance with manuring rate, providing an indication of the contribution of this practice to cultivation intensity. By combining weed ecology with crop isotope measurements, it is possible to identify anomalous δ^15^N values that do not relate to manuring, nor reflect agricultural productivity.

Further work is needed to extend this approach beyond temperate Europe, incorporating present-day studies of traditional farming in more arid regions, where water becomes limiting for crop growth and manuring is more strategically practiced depending on sufficient water availability (cf. Halstead 2014).

Preliminary application of the new combined ‘weed + isotope’ model to the investigation of cereal cultivation intensity in Neolithic central Europe suggests that early farmers in the region aimed to achieve highly productive conditions, in part through manuring, but that management intensity was variable. The Neolithic regime appears largely ‘intermediate’ between the high- and low-intensity extremes of Asturias and Haute Provence, respectively. This relative position is plausible, since neither of the modern studies represents a genuine ‘subsistence’ system: spelt production in Asturias was on a very restricted scale for occasional consumption or as a minor cash crop, while the Haute Provence system represents commercial organic production. It is likely that Neolithic farmers took a flexible approach to land management in response to changing availability of labour, manure etc. as well as shifting demands for sharing and hospitality (Halstead 2014). An intriguing implication of the range of variation in cultivation intensity revealed here is that agricultural productivity varied considerably. Integrated analysis of bioarchaeology and material culture is needed to understand how such productive inequalities were managed socially in local communities (e.g. Bogaard et al. 2011).

Our preliminary analysis of Neolithic central European cultivation intensity in light of the new model suggests that general theories of agricultural ‘intensification’ or ‘extensification’ in prehistory are likely too simplistic. A nuanced understanding of the historical development of farming practice will demand scrutiny of well documented regional sequences in order to track the ways in which cropping regimes shaped and responded to the wider (social) ecology.

## Electronic supplementary material

Below is the link to the electronic supplementary material.

Supplementary material 1 (PDF 38 kb)

## References

[CR1] Araus JL, Febrero A, Buxó R, Rodriguez-Ariza MO, Molina F, Camalich MD, Martin D, Voltas J (1997). Identification of ancient irrigation practices based on the carbon isotope discrimination of plant seeds: a case study from the south-east Iberian Peninsula. J Archaeol Sci.

[CR2] Araus JL, Febrero A, Catala M, Molist M, Voltas J, Romagosa I (1999). Crop water availability in early agriculture: evidence from carbon isotope discrimination of seeds from a tenth millennium BP site on the Euphrates. Glob Change Biol.

[CR3] Billy C, Billen G, Sebilo M, Birgand F, Tournebize J (2010). Nitrogen isotopic composition of leached nitrate and soil organic matter as an indicator of denitrification in a sloping drained agricultural plot and adjacent uncultivated riparian buffer strips. Soil Biol Biochem.

[CR4] Blondel J, Aronson J, Bodiou J-Y, Boeuf G (2010). The mediterranean region: biological diversity through time and space.

[CR5] Bogaard A (2002). Questioning the relevance of shifting cultivation to neolithic farming in the loess belt of western-central Europe: evidence from the Hambach Forest experiment. Veget Hist Archaeobot.

[CR6] Bogaard A (2004). Neolithic farming in central Europe.

[CR7] Bogaard A (2012). Plant use and crop husbandry in an early neolithic village: Vaihingen an der Enz, Baden-Württemberg.

[CR8] Bogaard A (2015) Lessons from modeling Neolithic farming practice: methods of elimination. In: Chapman R, Wylie A (eds) Material culture as evidence. Routledge, London, pp 243–254

[CR9] Bogaard A, Palmer C, Charles M, Jones G, Hodgson JG (1999). A FIBS approach to the use of weed ecology for the archaeobotanical recognition of crop rotation regimes. J Archaeol Sci.

[CR10] Bogaard A, Jones G, Charles M, Hodgson JG (2001). On the archaeobotanical inference of crop sowing time using the FIBS method. J Archaeol Sci.

[CR11] Bogaard A, Heaton THE, Poulton P, Merbach I (2007). The impact of manuring on nitrogen isotope ratios in cereals: archaeological implications for reconstruction. J Archaeol Sci.

[CR12] Bogaard A, Krause R, Strien H-C (2011). Towards a social geography of cultivation and plant use in an early farming community: Vaihingen an der Enz, south-west Germany. Antiquity.

[CR13] Bogaard A, Fraser R, Heaton THE (2013). Crop manuring and intensive land management by Europe’s first farmers. Proc Natl Acad Sci.

[CR14] Bol R, Eriksen J, Smith P, Garnett MH, Coleman K, Christensen BT (2005). The natural abundance of C-13, N-15, S-34 and C-14 in archived (1923-2000) plant and soil samples from the Askov long-term experiments on animal manure and mineral fertilizer. Rapid Commun Mass Spectrom.

[CR15] Charles M, Hoppé C (2003). The effects of irrigation on the weed floras of winter cereal crops in Wadi Ibn Hamad (southern Jordan). Levant.

[CR16] Charles M, Jones G, Hodgson JG (1997). FIBS in archaeobotany: functional interpretation of weed floras in relation to husbandry practices. J Archaeol Sci.

[CR17] Charles M, Bogaard A, Jones G, Hodgson J, Halstead P (2002). Ecological investigation of intensive cereal cultivation in the mountains of Asturias, NW Spain. Veget Hist Archaeobot.

[CR18] Charles M, Hoppé C, Jones G, Bogaard A, Hodgson J (2003). Using weed functional attributes for the identification of irrigation regimes in Jordan. J Archaeol Sci.

[CR19] Choi WJ, Ro HM, Hobbie EA (2003). Patterns of natural N-15 in soils and plants from chemically and organically fertilized uplands. Soil Biol Biochem.

[CR20] Condon AG, Richards RA, Farquhar GD (1987) Carbon isotope discrimination is positively correlated with grain yield and dry matter production in field-grown wheat. Crop Sci 27:996–1,001

[CR21] Dale JE (1982). The growth of leaves.

[CR22] Ellenberg H, Weber HE, Düll R, Wirth V, Werner W, Paulissen D (1992). Zeigerwerte von Pflanzen in Mitteleuropa. Scr Geobot.

[CR23] Ertuğ F (2004). Recipes of old tastes with einkorn and emmer wheat. TÜBA-AR.

[CR24] Farquhar GD, Richards RA (1984). Isotopic composition of plant carbon correlates with water-use efficiency of wheat genotypes. Aust J Plant Physiol.

[CR25] Ferrio JP, Voltas J, Alonso N, Araus JL, Dawson TE, Siegwolf R (2007). Reconstruction of climate and crop conditions in the past based on the carbon isotope signature of archaeobotanical remains. Isotopes as indicators of ecological change.

[CR26] Fraser R, Bogaard A, Heaton T, Charles M, Jones G, Christensen BT, Halstead P, Merbach I, Poulton PR, Sparkes D, Styring A (2011). Manuring and stable nitrogen isotope ratios in cereals and pulses: towards a new archaeobotanical approach to the inference of land use and dietary practices. J Archaeol Sci.

[CR27] Gilman A (1981). The development of social stratification in bronze age Europe. Curr Anthropol.

[CR28] Givnish TJ (1987). Comparative studies of leaf form: assessing the relative roles of selective pressures and phylogenetic constraints. New Phytol.

[CR29] Grime JP (1979). Plant strategies and vegetation processes.

[CR30] Grime JP, Hodgson JG, Hunt R (1988). Comparative plant ecology: a functional approach to common British species.

[CR31] Hajnalová M, Dreslerová D (2010). Ethnobotany of einkorn and emmer in Romania and Slovakia: towards interpretation of archaeological evidence. Památ Archeol.

[CR32] Halstead P (1995). Plough and power: the economic and social significance of cultivation with the ox-drawn ard in the Mediterranean. Bull Sumer Agric.

[CR33] Halstead P, Serjeantson D, Field D (2006). Sheep in the garden: the integration of crop and livestock husbandry in early farming regimes of Greece and southern Europe. Animals in the Neolithic of Britain and Europe.

[CR34] Halstead P (2014) Two oxen ahead: pre-mechanized farming in the Mediterranean. Wiley, Chichester

[CR35] Heaton THE, Jones G, Halstead P, Tsipropoulos T (2009) Variations in the 13C/12C ratios of modern wheat grain, and implications for interpreting data from Bronze Age Assiros Toumba, Greece. J Archaeol Sci 36:2224–2233

[CR36] Jackson LWR (1967). Effect of shade on leaf structure of deciduous tree species. Ecology.

[CR37] Jones G (2005). Garden cultivation of staple crops and its implications for settlement location and continuity. World Archaeol.

[CR38] Jones G, Charles M, Colledge S, Halstead P, Kroll H, Pasternak R (1995). Towards the archaeobotanical recognition of winter-cereal irrigation: an investigation of modern weed ecology in northern Spain. Res archaeobotanicae.

[CR39] Jones G, Bogaard A, Halstead P, Charles M, Smith H (1999). Identifying the intensity of crop husbandry practices on the basis of weed floras. Annu Br Sch Athens.

[CR40] Jones G, Bogaard A, Charles M, Hodgson JG (2000). Distinguishing the effects of agricultural practices relating to fertility and disturbance: a functional ecological approach in archaeobotany. J Archaeol Sci.

[CR41] Jones G, Charles M, Bogaard A, Hodgson JG (2010) Crops and weeds: the role of weed functional ecology in the identification of crop husbandry methods. J Archaeol Sci 37:70–77

[CR42] Kanstrup M, Thomsen IK, Mikkelsen PH, Christensen BT (2012). Impact of charring on cereal grain characteristics: linking prehistoric manuring practice to δ^15^N signatures in archaeobotanical material. J Archaeol Sci.

[CR43] Karagöz A, Padulosi S, Hammer K, Heller J (1996). Agronomic practices and socioeconomic aspects of emmer and einkorn cultivation in Turkey. Hulled wheats: promoting the conservation and use of underutilized and neglected crops 4.

[CR44] Kragten J (1994). Calculating standard deviations and confidence intervals with a universally applicable spreadsheet technique. Analyst.

[CR45] Moles AT, Warton DI, Warman L (2009). Global patterns in plant height. J Ecol.

[CR46] Nitsch EK, Charles M, Bogaard A (2015) Calculating a statistically robust δ^13^C and δ^15^N offset for charred cereal and pulse seeds. STAR, 1(1). STAR20152054892315Y.0000000001

[CR47] Palmer C (1998). An exploration of the effects of crop rotation regime on modern weed floras. Environ Archaeol.

[CR48] Poorter H, Niinemets U, Poorter L, Wright IJ, Villar R (2009). Causes and consequences of variation in leaf mass per area (LMA): a meta-analysis. New Phytol.

[CR49] Reich PB (1993). Reconciling apparent discrepancies among studies relating life span, structure and function of leaves in contrasting plant life forms and climates: ‘the blind men and the elephant retold’. Funct Ecol.

[CR50] Reich PB, Walters MB, Ellsworth DS (1992). Leaf life-span in relation to leaf plant and stand characteristics among diverse ecosystems. Ecol Monogr.

[CR51] Rothmaler W (1995). Exkursionsflora von Deutschland 3.

[CR52] Sans FX, Masalles RM (1995). Phenological patterns in an arable land weed community related to disturbance. Weed Res.

[CR53] Sherratt A (1997). Economy and society in prehistoric Europe.

[CR54] Szpak P (2014) Complexities of nitrogen isotope biogeochemistry in plant-soil systems: implications for the study of ancient agricultural and animal management practices. Front Plant Sci, 5. Article no. 288 10.3389/fpls.2014.00288 PMC4066317 25002865

[CR55] Ter Braak CFJ, Smilauer P (2002). Canoco reference manual and Canodraw for windows user’s guide (version 4.5).

[CR56] Trigger B (2003). Understanding early civilizations: a comparative approach.

[CR57] Wallace M, Jones G, Charles M, Fraser R, Halstead P, Heaton THE, Bogaard A (2013). Stable carbon isotope analysis as a direct means of inferring crop water status and water management practices. World Archaeol.

[CR58] Wright IJ, Reich PB, Westoby M (2004). The worldwide leaf economics spectrum. Nature.

