# Supplementary material for: Combining functional weed ecology and crop stable isotope ratios to identify cultivation intensity: a comparison of cereal production regimes in Haute Provence, France and Asturias, Spain
Source: Veg Hist Archaeobot. 2015 Mar 19;25(1):57–73. doi: 10.1007/s00334-015-0524-0 (PMC4709954; doi:10.1007/s00334-015-0524-0)
Supplement: Supplementary file 1 — Supplementary material 1 (PDF 38 kb) [file 334_2015_524_MOESM1_ESM.pdf]

Combining functional weed ecology and crop stable isotope ratios to identify cultivation intensity: a comparison of cereal production regimes in Haute Provence, France and Asturias, Spain

AMY BOGAARD*, JOHN HODGSON, ERIKA NITSCH, GLYNIS JONES, AMY STYRING, CHARLOTTE DIFFEY, JOHN POUNCETT, CHRISTOPH HERBIG, MIKE CHARLES, FÜSUN ERTUĞ, OSMAN TUGAY, DRAGANA FILIPOVIC, REBECCA FRASER

*Corresponding author contact details: School of Archaeology, University of Oxford, 36 Beaumont Street, Oxford OX PG, United Kingdom

Electronic Supplementary Material

Supplementary Table 1. Weed species recorded in at least 5% (3 or more) of the 60 crop field transects in Haute Provence

Species name
Aegilops cylindrica
Agrostemma githago
Ajuga chamaepitys ssp. *chamaepitys*
Alopecurus myosuroides
Alyssum alyssoides
Anagallis arvensis ssp. *caerulea*
Androsace maxima
Anthemis arvensis
Arenaria serpyllifolia
Avena sterilis
Bifora radians
Anisantha sterilis (= *Bromus sterilis*)
Lithospermum arvense (= *Buglossoides arvensis*)
Bupleurum rotundifolium
Camelina rumelica
Capsella bursa-pastoris
Centaurea cyanus
Centaurea scabiosa
Centaurea solstitialis
Cerastium pumilum
Chenopodium album
Chondrilla juncea
Cirsium arvense
Consolida pubescens
Consolida regalis
Convolvulus arvensis
Daucus carota ssp. *carota*
Euphorbia falcata
Fallopia convolvulus (= *Polygonum convolvulus*)
Galium aparine
Galium parisiense
Galium tricornutum
Geranium columbinum
Holosteum umbellatum
Kickxia spuria
Knautia integrifolia
Lactuca serriola
Lamium amplexicaule
Legousia speculum-veneris
Lepidium campestre
Lolium rigidum
Medicago lupulina
Medicago sativa ssp. *sativa*
Melilotus officinalis
Myagrum perfoliatum
Neslia paniculata
Orlaya daucoides
Papaver argemone
Papaver rhoeas
Picris hieracioides
Plantago lanceolata
Poa compressa
Polygonum aviculare
Potentilla reptans
Ranunculus arvensis
Rapistrum rugosum
Reseda phyteuma
Rubus caesius
Rumex crispus
Sanguisorba minor
Silene vulgaris
Thlaspi perfoliatum
Tordylium maximum
Torilis arvensis
Trifolium arvense
Trifolium pratense
Trigonella esculenta
Vaccaria hispanica
Veronica arvensis
Veronica hederifolia
Veronica persica
Veronica praecox
Vicia pannonica
Vicia sativa ssp. *sativa*
Vicia tenuifolia
Viola arvensis
Viola kitaibeliana
Vulpia unilateralis

Supplementary Table 2 Summary of Neolithic archaeobotanical samples included in the weed ecological analysis; see Supplementary References for full citation of data sources

Site	Period	No. samples	Reference
Aiterhofen	EN (LBK)	1	Bakels 1983/4
Aldingen	LN (Schwieberdingen)	1	Piening 1986a, 1992
Altdorf	EN (LBK)	1	Bakels 1983/4
Bedburg-Garsdorf/Bergheim	EN (LBK)	3	Knörzer 1974
Bruchenbrücken	EN (LBK)	2	Kreuz 1990
Ditzingen	EN (LBK)	3	Piening 1998
Ecsegfalva	EN (Körös)	1	Bogaard et al. 2007
Endersbach	MN (Großgartach)	1	Piening 1982
Erbedingen-Hochdorf	LN (Schussenried)	11	Küster 1985
Grossachsenheim	LN (Schussenried)	1	Piening 1986b
Hienheim/Donau	EN (LBK)	1	Bakels 1978, 1986
Hilzingen, Kr. Konstanz	EN (LBK)	4	Stika 1991
Hornstaad-Hörnle	LN (Hornstaad)	1	Maier 2001
Iwanowice-Klin	MN (Lengyel)	1	Litynska 1990
Kamenin	LN (Baden)	1	Nevizansky 1980; Hajnalová 1989
Lamersdorf/Düren	EN (LBK)	1	Knörzer 1967, 1968, 1971
Langweiler 16	EN (LBK)	1	Knörzer 1997
Langweiler 2/Jülich	EN (LBK)	1	Knörzer 1973
Langweiler 3/Jülich	EN (LBK)	1	Knörzer 1972
Langweiler 8/Jülich	EN (LBK)	10	Knörzer 1988
Langweiler 9/Jülich	EN (LBK)	2	Knörzer 1977
Laurenzberg 7	EN (LBK)	5	Knörzer 1997
Laurenzberg 8	EN (LBK)	1	Knörzer 1997
Maastricht-Randwijck	MN (Rössen)	1	Bakels 1991a; Bakels et al. 1993
Meckenheim/Bonn	EN (LBK)	2	Knörzer 1967, 1968, 1971
Meindling	EN (LBK)	3	Bakels 1992b
Mythenschloss	LN (Corded Ware)	1	Jacomet et al. 1989, unpubl. data; Brombacher and Jacomet 1997
Ulm-Eggingen	EN (LBK)	6	Gregg 1989
Vaihingen/Enz	EN (LBK)	71	Bogaard 2012
Wanlo/Wickerath	EN (LBK)	2	Knörzer 1980

Supplementary References

Bakels CC (1978) Four Linearbandkeramik settlements and their environment: a palaeoecological study of Sittard, Stein, Elsloo and Hienheim. Analecta Praehistorica Leidensia 11

Bakels CC (1983/4) Pflanzenreste aus Niederbayern - Beobachtungen in rezenten Ausgrabungen. Bericht der Bayerischen Bodendenkmalpflege 24/25:157-66

Bakels CC (1986) Früchte und Samen. In: Modderman PJR (ed) Die neolithische Besiedlung bei Hienheim, Lkr. Hienheim II-IV. Materialhefte zur Bayerischen Vorgeschichte A 57, pp 68-75

Bakels CC (1991) The crops of the Rössen culture. In: Vytlacok S (ed) Palaeoethnobotany and Archaeology: International Work-group for Palaeoethnobotany 8th Symposium Nitra-Nove Vozokany 1989. Nitra: Archaeological Institute of the Slovak Academy of Sciences, pp 23-7

Bakels CC, Alkemade MJ, Vermeeren CE (1993) Botanische Untersuchungen in der Rössener Siedlung Maastricht-Randwijck. In: Kalis AJ, Meurers-Balke J (eds) 7000 bäuerliche Landschaft: Entstehung, Erforschung, Erhaltung. Zwanzig Aufsätze zu Ehren von Karl-Heinz Knörzer. Cologne: Rheinland-Verlag GmbH, pp 35-48

Bakels CC (1992) The botanical shadow of two early Neolithic settlements in Belgium: carbonized seeds and disturbances in a pollen record. Review of Palaeobotany and Palynology 73:1-19

Bogaard A (2012) Plant Use and Crop Husbandry in an Early Neolithic Village: Vaihingen an der Enz, Baden-Württemberg. Frankfurter Archäologische Schriften. Bonn, Habelt-Verlag

Bogaard A, Bending J, Jones G (2007) Archaeobotanical evidence for plant husbandry and use at Ecsegfalva 23. In: Whittle A (ed) The Early Neolithic on the Great Hungarian Plain: investigations of the Körös culture site of Ecsegfalva 23, Co. Békés. Budapest, Hungarian Academy of Sciences, pp 421-445

Gregg SA (1989) Paleo-ethnobotany of the Bandkeramik phases. In: Kind C-J (ed) Ulm-Eggingen: Die Ausgrabungen 1982 bis 1985 in der bandkeramischen Siedlung und der mittlalterischen Wüstung. Forschungen und Berichte zur Vor- und Frühgeschichte in Baden-Württemberg 34, pp 367-99

Jacomet S, Brombacher C, Dick M (1989) Archäobotanik am Zürichsee. Ackerbau, Sammelwirtschaft und Umwelt von neolithischen und bronzezeitlichen Seeufersiedlungen im Raum Zürich. Zurich: Orell Füssli Verlag

Brombacher C, Jacomet S (1997) Ackerbau, Sammelwirtschaft und Umwelt: Ergebnisse archäobotanischer Untersuchungen. In: Schibler J, Hüster-Plogmann H, Jacomet S, Brombacher C, Gross-Klee E, Rast-Eicher A (eds) Ökonomie und Ökologie neolithischer und bronzezeitlicher Ufersiedlungen am Zürichsee. Zurich, Zürich und Egg, pp 220-91

Knörzer K-H (1967) Subfossile Pflanzenreste von bandkeramischen Fundstellen im Rheinland. Archaeo-Physika 2:3-29

Knörzer K-H (1968) 6000jährige Geschichte der Getreidenahrung im Rheinland. Decheniana 119:113-24

Knörzer K-H (1971) Urgeschichtliche Unkräuter im Rheinland: ein Beitrag zur Entstehungsgeschichte der Segetalgesellschaften. Vegetatio, 23:89-111

Knörzer K-H (1972) Subfossile Pflanzenreste aus der bandkeramischen Siedlung Langweiler 3 und 6, Kreis Jülich, und ein urnenfelderzeitlicher Getreidefund innerhalb dieser Siedlung. Bonner Jahrbücher 172:395-403

Knörzer K-H (1973) Pflanzliche Großreste. Rheinische Ausgrabungen 13: 139-52

Knörzer K-H (1974) Bandkeramische Pflanzenfunde von Bedburg-Garsdorf, Kreis Bergheim/Erft. Rheinische Ausgrabungen 15:173-92

Knörzer K-H (1977) Pflanzliche Großreste des bandkeramischen Siedlungsplatzes Langweiler 9. Rheinische Ausgrabungen 18: 279-303

Knörzer K-H (1980) Pflanzliche Großreste des bandkeramischen Siedlungsplatzes Wanlo (Stadt Möchengladbach). Archaeo-Physika 7:7-20

Knörzer K-H (1988) Untersuchungen der Früchte und Samen. In: Boelicke U, von Brandt D, Lüning J, Stehli P, Zimmerman A (eds) Der bandkeramische Siedlungsplatz Langweiler 8, Gemeinde Aldenhoven, Kreis Düren. Cologne, Rheinland-Verlag GmbH, pp 813-52

Knörzer K-H (1997) Botanische Untersuchung von 16 neolithischen Siedlungsplätzen im Bereich der Aldenhovener Platte, Kr. Düren und Aachen. In: Lüning J (ed) Studien zur neolithischen Besiedlung der Aldenhovener Platte und ihrer Umgebung. Cologne, Rheinland-Verlag GmbH, pp 647-84

Kreuz A (1990) Die ersten Bauern Mitteleuropas - eine archäobotanische Untersuchung zur Umwelt und Landwirtschaft der Ältesten Bandkeramik. Analecta Praehistorica Leidensia 23

Küster H (1985) Neolithische Pflanzenreste aus Hochdorf, Gemeinde Eberdingen (Kreis Ludwigsburg). In: Küster H, Körber-Grohne, U (eds) Horchdorf I. Forschungen und Berichte zur Vor- und Frühgeschichte in Baden-Württemberg 19, pp 13-83

Litynska M (1990) Zboza i chwasty z neolitycznego stanowiska Iwanowice-Klin, woj. Krakow. Sprawozdania Archeologiczne 62:105-8

Maier U (2001) Botanische und pedologische Untersuchungen zur Ufersiedlung Hornstaad-Hörnle IA. Stuttgart, Konrad Theiss Verlag

Nevizansky G (1980) Zachranny vyskum v Kamenine. Archeologicke vyskumy a nalezy na Slovensku v roku 1978:187-8

Hajnalová E (1989) Katalóg zvyskov semien a plodov v archeologickych nálezoch na Slovensku. Acta Interdisciplinaria Archeologica 6:3-192

Piening U (1982) Botanische Untersuchungen an verkohlten Pflanzenresten aus Nordwürttemberg: Neolitische bis römische Zeit. Fundberichte aus Baden-Württemberg 7:39-71

Piening U (1986a) Verkohlte Getreidevorräte von Aldingen, Gem. Remseck am Neckar, Kreis Ludwigsburg. Fundberichte aus Baden-Württemberg 11, 191-208

Piening U (1986b) Verkohlte Nutz- und Wildpflanzenreste aus Grossachsenheim, Gem. Sachsenheim, Kreis Ludwigsburg. Fundberichte aus Baden-Württemberg 11:177-90

Piening U (1992) Nutzpflanzenreste der Schussenrieder Kultur von Aldingen, Kreis Ludwigsburg. Fundberichte aus Baden-Württemberg 17:125-42

Piening U (1998) Die Pflanzenreste aus Gruben der Linearbandkeramik und der Rössener Kultur von Ditzingen, Kr. Ludwigsburg. Fundberichte aus Baden-Württemberg 22:125-60

Stika H-P (1991) Die paläoethnobotanische Untersuchung der linearbandkeramischen Siedlung Hilzingen, Kreis Konstanz. Fundberichte aus Baden-Württemberg 16:63-104
